# Comparing Stenting with Medical Therapy Versus Medical Therapy Alone in Patients with Intracranial Atherosclerotic Stenosis: A Current Systematic Review and Meta-Analysis

**DOI:** 10.3390/clinpract15060113

**Published:** 2025-06-19

**Authors:** Khalid Bin Aziz, Hussam Alhathlol, Fahad Bin Aziz, Mohammed Alshammari, Mohammed Ali Alhefdhi, Abdulrahman M. Alrasheed, Nawwaf Alfayez, Thamer S. Alhowaish

**Affiliations:** 1 College of Medicine, King Saud bin Abdulaziz University for Health Sciences, Riyadh 11481, Saudi Arabia; 2 King Abdullah International Medical Research Center, Riyadh 11481, Saudi Arabia; 3 Department of Neurology, King Abdulaziz Medical City, Ministry of the National Guard Health Affairs (MNGHA), Riyadh 11481, Saudi Arabia

**Keywords:** ischemic stroke, percutaneous transluminal angioplasty, stenting, symptomatic intracranial arterial stenosis

## Abstract

**Background:** Intracranial atherosclerotic stenosis (ICAS) is a significant cause of ischemic stroke worldwide, with high recurrence rates despite optimal medical therapy. While endovascular stenting has been proposed as an adjunctive treatment, its clinical benefit remains controversial as a first line therapy. **Objective:** To evaluate the efficacy and safety of stenting plus medical therapy (STN+MT) compared to medical therapy alone (MT) in patients with symptomatic ICAS through a systematic review and meta-analysis of randomized controlled trials (RCTs). **Methods**: We systematically searched PubMed, Web of Science, the Cochrane Library, Embase, Scopus, and EBSCO for RCTs comparing STN+MT with MT in adult patients with symptomatic ICAS. Primary outcomes included transient ischemic attack (TIA), stroke, intracerebral hemorrhage (ICH), and death at 30 days and 1 year. Pooled risk ratios with 95% confidence intervals were calculated using random-effects or fixed-effects models as appropriate. Meta-regression was conducted to assess effect modification by study-level characteristics. **Results**: Four trials comprising 990 patients were included. STN+MT was associated with significantly higher 30-day risk of stroke and ICH compared to MT alone. No significant differences in TIA, stroke, ICH, or death were found at 1 year. Meta-regression revealed no significant effect modifiers, suggesting consistent findings across subgroups. **Conclusions**: Our meta-analysis consolidates the evidence that intracranial stenting as a first line therapy offers no significant advantage over medical therapy in preventing stroke in symptomatic ICAS, while it does pose added early risks. This holds true across different trials, patient demographics, and clinical scenarios examined. The consistency of this message across multiple RCTs provides a high level of evidence to guide practice. At present, aggressive medical therapy alone should be the default management for most patients. Endovascular intervention should be reserved for clinical trial settings or carefully selected salvage cases, until and unless new evidence emerges to change the risk–benefit calculus such as the promising use of balloon angioplasty in the BASIS trial.

## 1. Introduction

Stroke remains a leading cause of death and long-term disability worldwide. According to the World Health Organization, stroke is consistently among the top three causes of death globally, with ischemic stroke accounting for the majority of cases [1]. The burden of stroke is particularly significant in low- and middle-income countries, where access to timely interventions remains limited [2]. Intracranial atherosclerotic stenosis (ICAS) is a major cause of ischemic stroke, especially among Asian, Black, and Hispanic populations [3]. Patients with high-grade stenosis (≥70%) who have experienced recent ischemic events, such as stroke or transient ischemic attack, are at especially high risk for recurrence [4]. ICAS is estimated to account for up to 10% of all ischemic strokes in Western countries and as much as 30–50% in some Asian populations, and a single retrospective study in Saudi Arabia found that it is the mechanism of 26% of ischemic stroke cases [5,6,7]. Conventional vascular risk factors—including hypertension, diabetes mellitus, dyslipidemia, smoking, and metabolic syndrome—strongly predispose individuals to ICAS. Chronic hypertension, in particular, causes endothelial injury and hemodynamic stress, and intracranial arteries (with their thinner media and sparse elastic fibers) are uniquely susceptible to hypertensive damage making them prone to developing atherosclerotic stenosis [8]. These influences contribute to the development of ICAS and partly explain its epidemiological pattern and ethnic disparities.

**Figure 1:**
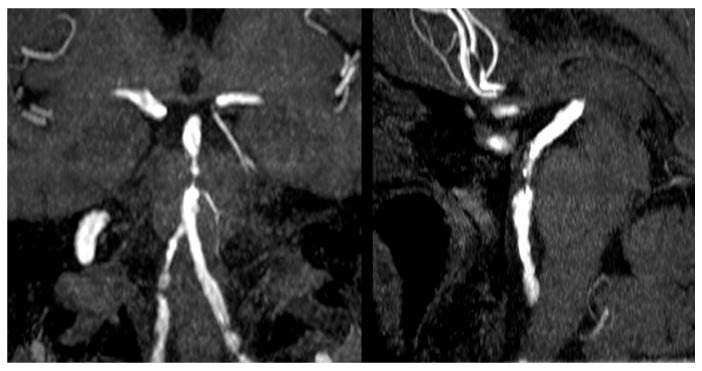
Coronal and sagittal MRA showing multiple stenosis points on vertebrobasilar system. Case courtesy of Bruno Di Muzio, https://radiopaedia.org/?lang=gb (accessed on 5 June 2025) (Radiopaedia.org). From the case https://radiopaedia.org/cases/30288?lang=gb (accessed on 5 June 2025) (rID: 30288) [11].

ICAS is characterized by a chronic inflammatory atherosclerotic process in large intracranial arteries, often at sites of high shear stress (e.g., arterial bifurcations and curvatures). Endothelial dysfunction and the accumulation of oxidized LDL in the arterial intima trigger a cascade of monocyte infiltration, foam cell formation, and plaque development, with a lipid-rich necrotic core and a thinning fibrous cap. Advanced plaques in intracranial vessels frequently exhibit neovascularization from the vasa vasorum, predisposing them to intraplaque hemorrhage and plaque instability. Once significant stenosis develops (Figure 1), ICAS can cause ischemic events through several mechanisms: (i) in situ thrombosis due to plaque rupture and occlusion of the artery, (ii) artery-to-artery embolization of thrombus distally, (iii) hemodynamic compromise from critical luminal narrowing, and (iv) branch occlusive disease where plaque encroachment occludes penetrating branch arteries [9]. Notably, ICAS-related strokes have among the highest recurrence rates of any stroke etiology—the one-year recurrent stroke risk exceeds 20% in patients with symptomatic severe ICAS despite medical therapy [10]. The high risk of stroke recurrence in ICAS highlights the need to explore treatments that help maintain intracranial arterial patency.

The management of symptomatic ICAS has traditionally centered on medical therapy, including antiplatelet agents, statins, blood pressure control, and lifestyle modifications. However, the high rate of recurrent strokes in this population has prompted investigations into alternative or adjunctive treatments. Endovascular therapy emerged in the 1980s as a potential treatment for patients with symptomatic intracranial atherosclerotic stenosis, particularly in cases where individuals with stenosis greater than 70% continue to experience ischemic events despite receiving optimal medical management [12]. To evaluate the role of stenting in patients with symptomatic intracranial stenosis, the first randomized controlled trial to directly compare stenting plus medical therapy versus medical therapy alone was the Stenting and Aggressive Medical Management for Preventing Recurrent Stroke in Intracranial Stenosis (SAMMPRIS) trial, conducted in 2011 [13]. Findings from both the SAMMPRIS trial and the Vitesse Intracranial Stent Study for Ischemic Stroke Therapy (VISSIT) trial demonstrated that medical therapy alone was more effective and safer than combining it with intracranial stenting [13,14]. However, these early trials had limitations, including high periprocedural complication rates, use of first-generation stents, and enrollment of patients in the early post-stroke phase when the vessel wall may be unstable. More recently, the Chinese Angioplasty and Stenting for Symptomatic Intracranial Severe Stenosis (CASSISS) trial re-examined the role of stenting under stricter procedural protocols and in experienced centers, reporting no significant difference in stroke or death rates between stenting and medical therapy, but demonstrating improved procedural safety compared to earlier trials [15]. Despite improved procedural safety, the CASSISS trial had several limitations: delayed enrollment that may have selected a more stable population with lower risk, exclusion of high-risk lesion locations, and reliance on highly experienced operators.

Given the heterogeneity of outcomes reported in major clinical trials, alongside continuous advancements in endovascular techniques and evolving criteria for patient selection, the optimal management strategy for symptomatic intracranial atherosclerotic stenosis (ICAS) remains a subject of ongoing debate. Although currently medical therapy is generally preferred, emerging evidence suggests that a subset of patients may derive significant benefit from endovascular intervention under carefully defined conditions. Therefore, this systematic review aims to critically evaluate the current evidence on the safety and efficacy of intracranial stenting compared to medical therapy in patients with symptomatic intracranial atherosclerotic stenosis.

## 2. Materials and Methods

**Figure 2:**
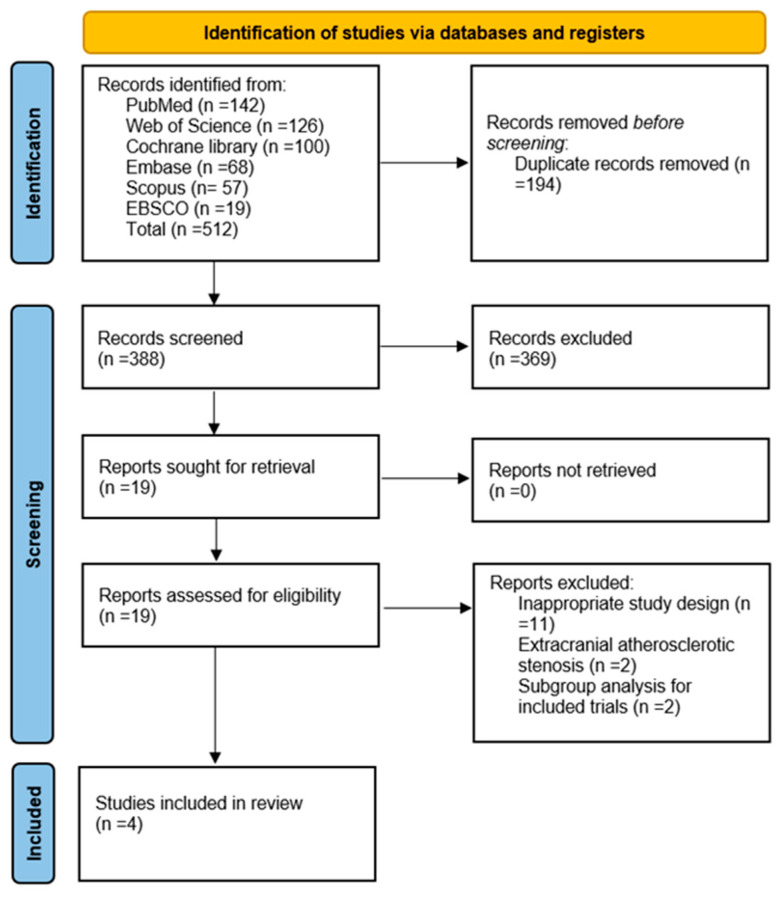
PRISMA flow chart presenting the selection of eligible studies.

This systematic review followed the Preferred Reporting Items for Systematic Reviews and Meta-Analyses (PRISMA) guidelines. The study protocol was registered in the National Institute of Health Research Prospective Register of Systematic Reviews PROSPERO (CRD420251022851). The study selection process is illustrated in the PRISMA flow diagram (Figure 2).

### 2.1. Information Sources and Search Strategy

A comprehensive search was conducted across the following databases: PubMed, Web of Science, the Cochrane Library, Embase, Scopus, and EBSCO. Specific search strategies employed for each database can be found in the Supplementary Materials (File S1). However, the search strategy utilized controlled vocabulary (e.g., MeSH terms) and relevant keywords: (Intracranial stenosis OR Intracranial atherosclerosis OR Cerebral artery stenosis) AND (Intracranial stenting OR Endovascular therapy OR Angioplasty OR Wingspan stent OR Balloon-expandable stent OR Self-expanding stent) AND (Medical therapy OR Aggressive medical management OR Dual antiplatelet therapy OR Aspirin and Clopidogrel) AND (Stroke OR Ischemic stroke OR Hemorrhagic stroke OR Mortality OR Restenosis) AND (Randomized controlled trial OR RCT).

### 2.2. Eligibility Criteria

This systematic review and meta-analysis included studies that met the following criteria: RCTs involving adult patients aged 18 years or older diagnosed with symptomatic intracranial atherosclerotic stenosis (ICAS) with a stenosis severity of 50–99%. Eligible studies must compare endovascular treatment with stenting plus optimal medical therapy to optimal medical therapy alone, which includes dual antiplatelet therapy and aggressive risk factor management. To be considered, studies must report at least one relevant clinical outcome, such as stroke (ischemic or hemorrhagic), transient ischemic attack (TIA), death, restenosis, or adverse procedural events. Only articles published in English were included, and a minimum follow-up duration of one month is required for eligibility.

Studies were excluded if they were non-randomized, including observational studies, case series, case reports, or review articles. Studies focusing exclusively on extracranial carotid stenosis rather than intracranial stenosis were also excluded. Additionally, studies that do not provide sufficient outcome data for analysis or that have a follow-up duration of less than one month were not included in this review. The detailed inclusion and exclusion criteria are summarized in Table 1.

### 2.3. Screening and Selection of Studies

All studies identified through database searches (PubMed, Web of Science, the Cochrane Library, Embase, Scopus, and EBSCO) were imported into an Excel spreadsheet for management and screening. Initially, two independent reviewers screened the titles and abstracts of all retrieved articles to identify potentially relevant studies. Any discrepancies between reviewers at this stage were resolved by consensus or referred for full-text evaluation.

Full-text articles of the selected studies were then independently assessed for eligibility by the same two reviewers based on the predefined inclusion and exclusion criteria. The references of the included articles were also manually screened to identify any additional eligible studies not captured in the initial search.

A standardized data extraction form was used to systematically extract relevant data from the full texts. Data extracted included: first author, year of publication, country of origin, sample size, mean or median patient age, sex distribution, degree of stenosis, affected vascular territory, type of intervention (including stent type), medical therapy regimen, follow-up duration, and reported outcomes.

Primary outcomes included stroke (ischemic or hemorrhagic), transient ischemic attack (TIA), or death within 30 days and one year. Discrepancies in study selection or data extraction were resolved through discussion, with arbitration by a third reviewer when necessary.

### 2.4. Risk of Bias Assessment

**Figure 3:**
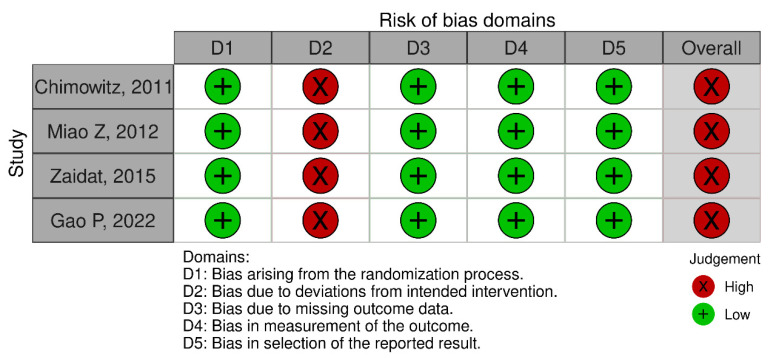
Risk of bias summary: review authors’ judgements about each risk of bias item for each included study [13,14,15,16].

**Figure 4:**
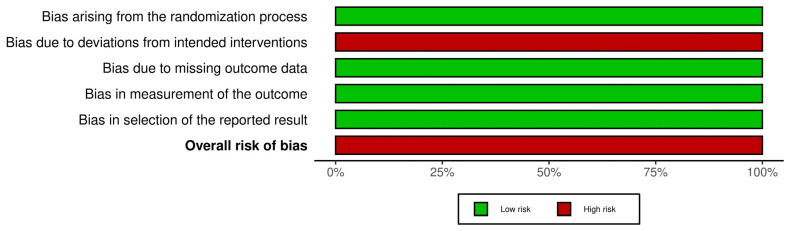
Risk of bias graph: percentage of included studies judged as low, unclear, or high risk of bias for each domain. In this analysis, all studies received the same judgment for each domain (e.g., 100% low risk or 100% high risk).

The risk of bias for each included RCT was assessed using the Cochrane Risk of Bias 2.0 (RoB 2) tool and is illustrated in Figure 3 and Figure 4. All included RCTs were judged to be at low risk of bias across the assessed domains, with the exception of domain D2 (deviations from intended interventions), which was uniformly high due to the inability to blind participants and personnel. Consequently, Figure 4 shows 100% values for each domain, indicating that all studies shared the same risk-of-bias rating per domain.

### 2.5. Statistical Analysis

All statistical analyses were conducted using RStudio (version 2024.9.1.394, Boston, MA, USA) with R version 4.4.2. For the meta-analysis, pooled risk ratios (RRs) and 95% confidence intervals (CIs) were calculated using the Mantel–Haenszel method. A common-effects model was applied when heterogeneity was low (I^2^ < 50%), while a random-effects model was used in cases of moderate-to-high heterogeneity. Statistical heterogeneity across studies was quantified using the I^2^ statistic. Publication bias was evaluated through visual inspection of funnel plots and formally tested using Egger’s test, where applicable. Meta-regression was performed to assess potential effect modifications by study-level covariates, but only for outcomes with sufficient data, as models requiring more parameters than available observations could not be fitted. A two-sided *p*-value < 0.05 was considered statistically significant.

## 3. Results

### 3.1. Characteristics of the Included Studies and Patients

Four RCTs were included, enrolling a total of 990 participants. The SAMMPRIS trial (USA) [13] had the largest sample size with 451 participants, followed by the CASSISS trial (China) [15] with 358 participants, the VISSIT trial (international) [14] with 111 participants, and the study by Miao et al. (China) [16] with 70 participants. The number of patients allocated to the stenting plus medical therapy (STN+MT, N = 489) and medical therapy alone (MT alone, N = 501) arms was nearly balanced across studies (49.39% and 50.61%, respectively). Males represented almost two-thirds of patients (N = 657, 66.36%). Mean ages ranged from 53.4 to 61.8 years in the STN+MT group and 49.2 to 61.8 years in the MT alone group. Wingspan stents were predominantly used in the SAMMPRIS [13] and CASSISS [15] trials. Miao et al. [16] used a variety of stents, including Wingspan, Coroflex Blue, and PTA Firebird stents. The VISSIT trial [14] utilized balloon-expanding stents exclusively (Table 2).

### 3.2. Baseline Clinical and Angiographic Characteristics of Participants

Across the four included trials, stroke was a more common cause of intervention than transient ischemic attack (TIA) in both treatment arms. In the SAMMPRIS trial [13], stroke was reported in 142 patients in the STN+MT group and 152 in the MT alone group, while TIA occurred in 82 and 75 patients, respectively. Similarly, in the CASSISS trial [15], stroke was reported in 89 and 105 patients and TIA in 87 and 77 patients in the STN+MT and MT alone groups, respectively. Data from the Miao and VISSIT trials showed smaller proportions with stroke and TIA [14,16].

Regarding arterial territory, the most frequently affected vessels in SAMMPRIS [13] were the middle cerebral artery (41.1%) and basilar artery (21.9%) in the STN+MT group. Comparable distributions were reported in the MT alone arm. In CASSISS [15], the middle cerebral artery (M1 segment) was the most commonly affected (36.9% in STN+MT and 43.4% in MT alone), followed by the basilar and vertebral arteries.

The time from qualifying event to randomization varied substantially between trials. The mean duration was shortest in SAMMPRIS [13] (9.0 ± 9.0 days for STN+MT and 6.7 ± 3.7 days for MT alone) and longest in the VISSIT trial [14] (12.3 ± 9.6 and 15.2 ± 10.3 days). In contrast, CASSISS [15] reported an intermediate duration with wide variability (42.3 ± 28.8 and 44.0 ± 29.9 days). Degree of arterial stenosis ranged from 78.4% to 85.0% across studies. Finally, the proportion of patients receiving antithrombotic therapy before the event was reported only in SAMMPRIS [13] (145 in STN+MT, 141 in MT alone) and CASSISS [15] (49 in STN+MT, 48 in MT alone, Table 3).

### 3.3. Death at 30 Days and 1 Year

**Figure 5:**
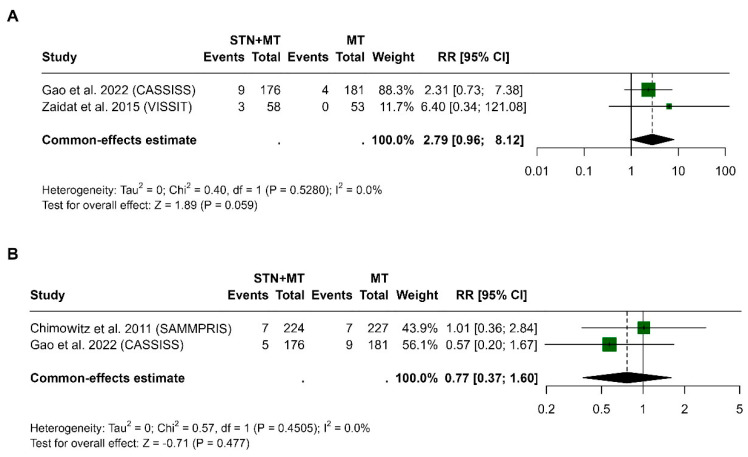
Forest plots comparing the risk ratios (RRs) of all-cause death between stenting plus medical therapy (STN+MT) and medical therapy alone (MT) at 30 days (**A**) and 1 year (**B**). Each study [13,14,15] is represented by a square (proportional to its weight in the analysis), and horizontal lines indicate 95% confidence intervals. The vertical line at RR = 1 represents no effect. Pooled estimates were calculated using a random-effects model.

Two studies were included for death at 30 days [14,15]. The pooled risk ratio (RR) was 2.79 (95% CI, 0.96 to 8.12) with an overall effect approaching statistical significance (*p* = 0.059). There was no significant heterogeneity across studies (I^2^ = 0%, *p* = 0.528, Figure 5A).

For the mortality at 1 year, including data from Chimowitz et al. 2011 (SAMMPRIS) [13] and Gao et al. 2022 (CASSISS) [15], the pooled risk ratio was 0.77 (95% CI, 0.37 to 1.60), indicating no significant difference between the treatment groups. Again, heterogeneity was negligible (I^2^ = 0%, *p* = 0.450), and the test for overall effect was not statistically significant (*p* = 0.477, Figure 5B).

### 3.4. Stroke at 30 Days and 1 Year

**Figure 6:**
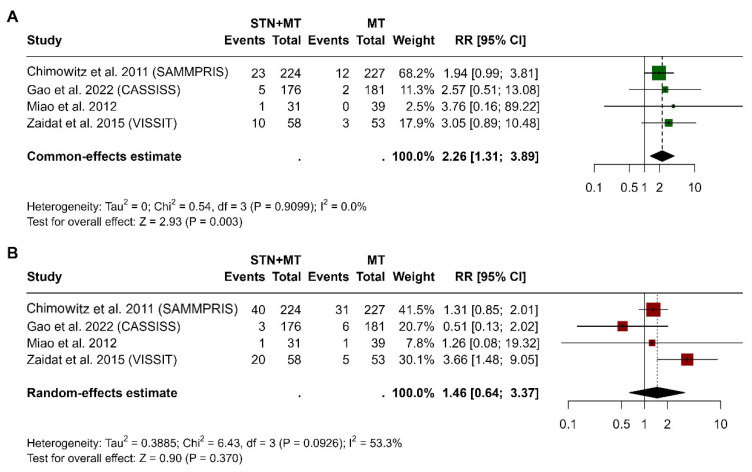
Forest plots showing the relative risk of stroke in the STN+MT group versus the MT group at 30 days (**A**) and at 1 year (**B**), based on data from the four randomized controlled trials [13,14,15,16]. Each square represents an individual trial’s effect estimate, with 95% confidence intervals shown as horizontal lines. The pooled risk ratio was significantly higher at 30 days but not at 1 year. The analysis was performed using a random-effects model.

In Figure 6, panel A shows the meta-analysis of 30-day stroke risk comparing STN+MT with MT alone across four studies [13,14,15,16]. The pooled risk ratio was 2.26 (95% CI, 1.31 to 3.89, *p* = 0.003), indicating a statistically significant increased risk in the STN+MT group. Heterogeneity was not significant (I^2^ = 0%, *p* = 0.911), suggesting consistency across studies. The 1-year stroke outcomes were from the same four studies. The pooled risk ratio was 1.46 (95% CI, 0.64 to 3.37, *p* = 0.370), indicating no statistically significant difference between the groups. Moderate heterogeneity was observed (I^2^ = 53.3%, *p* = 0.093), suggesting some variability in effect estimates across studies.

### 3.5. Intracerebral Hemorrhage at 30 Days and 1 Year

**Figure 7:**
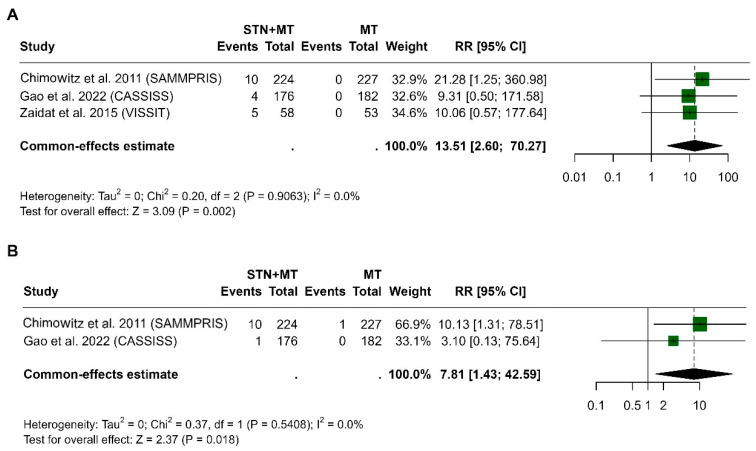
Forest plots of the risk of intracerebral hemorrhage (ICH) associated with stenting plus medical therapy compared to medical therapy alone, at 30 days (**A**) and 1 year (**B**), based on three randomized controlled trials [13,14,15]. Data are presented as risk ratios with 95% confidence intervals. The early periprocedural risk of ICH is notably higher in the STN+MT group. Results were pooled using a random-effects model.

At 30 days, the pooled risk ratio was 13.51 (95% CI, 2.60 to 70.27, *p* = 0.002), indicating a significantly higher risk in the STN+MT group compared to MT. No heterogeneity was observed (I^2^ = 0%, *p* = 0.907, Figure 7A). At 1 year, the pooled risk ratio was 7.81 (95% CI, 1.43 to 42.59, *p* = 0.018), again showing a significantly increased risk associated with STN+MT. Heterogeneity remained low (I^2^ = 0%, *p* = 0.541, Figure 7B).

### 3.6. Transient Ischemic Attack at 30 Days and 1 Year

**Figure 8:**
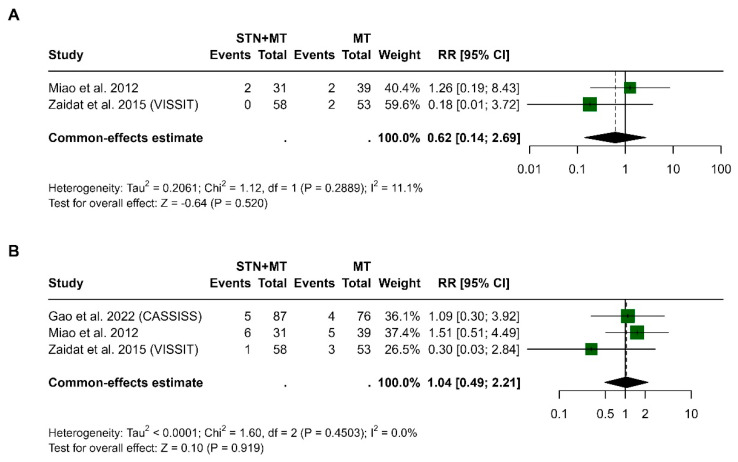
Forest plots comparing the incidence of transient ischemic attack (TIA) between STN+MT and MT groups at 30 days (**A**) and 1 year (**B**), based on the three trials reporting TIA outcomes [14,15,16]. Risk ratios and 95% confidence intervals are shown. No significant difference was observed in either time point. Data synthesis used a random-effects meta-analytic approach.

In Figure 8A, the results based on Miao et al., 2012 [16] and Zaidat et al., 2015 (VISSIT) [14] showed that the pooled risk ratio was 0.62 (95% CI, 0.14 to 2.69, *p* = 0.520), indicating no statistically significant difference between STN+MT and MT. Heterogeneity was low (I^2^ = 11.1%, *p* = 0.289). In Figure 8B, three studies—Gao et al., 2022 (CASSISS) [15], Miao et al., 2012 [16], and Zaidat et al., 2015 [14]—analyzed TIA at 1 year. The pooled risk ratio was 1.04 (95% CI, 0.49 to 2.21, *p* = 0.919), showing no significant difference in risk between treatment groups. No heterogeneity was detected (I^2^ = 0%, *p* = 0.450).

### 3.7. Publication Bias

**Figure 9:**
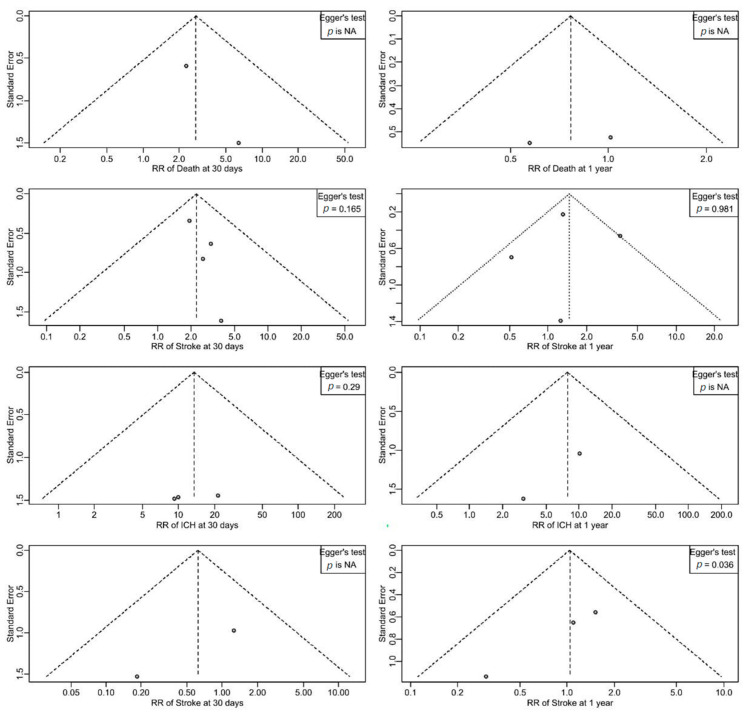
Funnel plots showing the risk of publication bias across different outcomes.

Funnel plots were used to assess publication bias across outcomes of death, stroke, ICH, and TIA at 30 days and 1 year (Figure 9). Visual inspection of the funnel plots revealed generally symmetrical distributions for most outcomes, suggesting a low likelihood of publication bias. Regarding the statistical assessment, Egger’s test was not applicable for death at 30 days and 1 year, ICH at 1 year, and TIA at 30 days due to the limited number of included studies. For stroke at 30 days, stroke at 1 year, and ICH at 30 days, Egger’s test indicated no significant evidence of publication bias, with *p*-values of 0.165, 0.981, and 0.290, respectively. However, for TIA at 1 year, Egger’s test suggested potential publication bias (*p* = 0.036).

### 3.8. Meta-Regression Analysis

Meta-regression was conducted only for selected outcomes due to limitations in data availability. Specifically, outcomes such as stroke at 30 days, stroke at 1 year, intracerebral hemorrhage at 30 days, and transient ischemic attack at 1 year were analyzed. The remaining outcomes were not included in the meta-regression because the number of parameters to be estimated exceeded the number of available studies, resulting in an error in model fitting. As shown in Table 4, none of the examined covariates—including age, year of publication, proportion of male participants, smoking status, stroke or TIA as the qualifying event, and degree of arterial stenosis—were significantly associated with any of the analyzed outcomes. All *p*-values were greater than 0.05, and the confidence intervals for the beta coefficients were wide, indicating no meaningful effect modification across these study-level variables.

## 4. Discussion

This systematic review and meta-analysis, encompassing four major randomized trials (SAMMPRIS, VISSIT, CASSISS, and a single-center Chinese trial by Miao et al.), provides a comprehensive comparison of intracranial stenting plus medical therapy (STN+MT) versus medical therapy alone (MT) for symptomatic intracranial atherosclerotic stenosis (ICAS). The pooled evidence confirms that adding percutaneous transluminal angioplasty and stenting (PTAS) to aggressive medical management does not improve long-term outcomes, but it does significantly increase early risks. Importantly, we observed a clear time-dependent pattern in treatment effects: at 30 days post-intervention, the risk of stroke (any type) was substantially higher in the stenting group than in the medical therapy group (pooled risk ratio ≈ 2.3, *p* = 0.003). This early excess risk with STN+MT is in line with the “early hazard” seen in each individual trial [17]. Periprocedural complications—especially intracerebral hemorrhages—were major contributors to this early disadvantage. For example, in the SAMMPRIS trial, 4.5% of patients receiving a stent suffered a symptomatic intracerebral hemorrhagic stroke within 30 days, whereas no such hemorrhages occurred in the medical arm. Consistently, our meta-analysis found the relative risk of intracerebral hemorrhage during the peri-procedural period to be dramatically elevated (on the order of a 13-fold increase) with stenting. These findings highlight that the initial procedural risk of stenting is considerable.

By the one-year follow-up, outcomes between STN+MT and MT had essentially converged. The pooled 1-year stroke risk was no longer significantly different between the two strategies (RR ≈ 1.46, 95% CI 0.64–3.37, *p* = 0.37). Rates of death and composite vascular events (including transient ischemic attacks) were likewise similar in both groups. In other words, any early harm from stenting was not offset by a later reduction in ischemic events—the initial risks of the invasive strategy yielded no net benefit by one year. Our results therefore reinforce each trial’s conclusion that aggressive medical therapy alone is as effective as (or superior to) stenting for preventing recurrent stroke at 12 months. In summary, adding PTAS to medical management provides no advantage in 1-year outcomes despite its higher 30-day complication rate, emphasizing that the early procedural risks of intracranial stenting outweigh any potential longer-term gains.

A new aspect of our analysis is the meta-regression exploring whether study-level patient characteristics modified the treatment effect. We found no significant interaction between the relative benefit of stenting (versus medical therapy) and factors such as mean age, sex distribution, baseline stenosis severity, timing of intervention, or smoking prevalence. This suggests the lack of efficacy of stenting is consistent across various subgroups and trial populations. Prior meta-analyses did not formally examine these effect modifiers, leaving open the question of whether certain patients (e.g., younger individuals, men vs. women, those with more severe stenosis, or those treated later after the stroke) might fare better with endovascular therapy [18,19,20,21]. Our results add confidence that no major subgroup derives clear benefit from stenting, at least within the ranges of characteristics examined across these trials. This finding is in line with a post hoc subgroup analysis of SAMMPRIS, which found no subset of patients (even those considered at particularly high risk on medical therapy) who benefited from stenting [17]. Similarly, a recent systematic review noted that delaying intervention beyond 3 weeks did not alter the overall lack of benefit of PTAS [21]. Together, these analyses show that the neutral or negative effect of stenting is still evident across different patient demographics and clinical profiles, reinforcing treatment effect consistency across subgroups.

It is important to interpret these findings in the context of the trial differences and other contemporary studies. The CASSISS trial notably differed from SAMMPRIS in its inclusion criteria regarding timing. CASSISS required patients to be beyond the hyperacute phase (no TIA/stroke within 3 weeks before enrollment), whereas SAMMPRIS enrolled patients as early as days after the qualifying event. This design choice in CASSISS likely excluded the highest-risk period for recurrent stroke, yielding markedly lower event rates in both arms compared to SAMMPRIS. Indeed, the 1-year stroke or death rate in the medically treated group was ~7% in CASSISS, much lower than ~12–15% in SAMMPRIS. With a more stable patient population and improved procedural safety, CASSISS reported a 30-day stroke/death rate around 2–3% for stenting—dramatically lower than the 14.7% seen in SAMMPRIS. Despite this, CASSISS still found no significant difference between stenting plus MT vs. MT Alone throughout 1 year. The different early findings can thus be largely explained by differences in timing and patient selection. By avoiding emergent post-stroke intervention, CASSISS demonstrated that intracranial stenting can be performed more safely; however, efficacy was not improved. Apart from timing, CASSISS also excluded certain high-risk anatomical scenarios (like perforator territory infarcts), and used the Wingspan stent with experienced operators, all of which likely contributed to safer procedure outcomes. These trial differences highlight the importance of design on outcomes; nonetheless, across both Western and Asian populations and varying protocols, no trial has shown a superiority of stenting over medical therapy.

In framing our results, it is instructive to discuss some relevant studies that were excluded from our analysis but provide additional insights. The WEAVE trial was a post-market registry (non-randomized) that assessed 152 patients treated with Wingspan stenting under the FDA’s on-label criteria (which mandated patients have failed medical therapy and are beyond 7 days from last stroke). WEAVE reported an impressively low 72 h stroke or death rate of only 2.6%—far below the complication rate in SAMMPRIS and even below CASSISS’s perioperative risk [22]. This indicates that in the most carefully selected patients (those who had multiple recurrent events despite medical therapy, and with the procedure performed by experienced interventionists under strict protocol), intracranial stenting can be performed with high safety. While encouraging from a safety standpoint, WEAVE did not have a control arm, so it cannot demonstrate efficacy. This suggests that procedural risks may be reduced, but it remains unproven whether stenting improves outcomes. A follow-up of that registry (WOVEN) suggested an 8.5% one-year stroke rate, which, although seemingly better than historical controls, must be interpreted with caution due to selection bias [23]. We excluded WEAVE because of its single-arm design but acknowledge that its findings provide a rationale for potentially revisiting randomized trials in a more selective fashion if any promise of benefit emerges.

Perhaps the most intriguing recent development is the BASIS trial (Balloon Angioplasty vs. Best Medical Therapy in Symptomatic ICAS) [24]. The BASIS trial, a randomized trial conducted in China, was excluded from our analysis because it tested balloon angioplasty without stenting rather than stent placement. Its findings, however, have significant implications for the broader role of endovascular therapy. BASIS is the first RCT to demonstrate a positive result for an endovascular approach in ICAS. Importantly, angioplasty in BASIS was performed in a controlled manner and after at least a few weeks of medical therapy. Even so, peri-procedural complications were not negligible: 14.5% of patients had an arterial dissection during angioplasty, and the majority of those required “bailout” stenting to seal the dissection. Despite these issues, the angioplasty group outcomes were superior to medical therapy, marking a stark contrast to prior stenting trials. How do we reconcile this? One hypothesis is that by foregoing stent implantation, the procedure avoids some risks such as long-term foreign body presence and stent thrombosis, while still alleviating the hemodynamic stenosis enough to reduce downstream ischemia. Furthermore, The BASIS investigators themselves acknowledge that their results will need replication in other populations, especially since over 85% of medically managed patients in BASIS still did not have a primary endpoint event at 1 year, highlighting that many patients do well on medical therapy alone. For now, BASIS provides a proof-of-concept that an endovascular strategy (specifically submaximal angioplasty) might confer benefit in ICAS, even though stenting has failed to do so. It opens the door to future trials comparing angioplasty, newer generation devices, or other novel techniques against best medical therapy. Our analysis focused on stenting trials; hence, BASIS was outside of our scope, but its implications are discussed here to acknowledge that the field is evolving. At the very least, BASIS suggests that the concept of endovascular intervention for intracranial stenosis should not be entirely written off—rather, it should be refined and tested in safer, more targeted ways.

For clinicians managing patients with symptomatic ICAS (≥50% stenosis and a recent ischemic event), the current synthesis of evidence indicates that aggressive risk factor management and dual antiplatelet therapy remain the cornerstone of treatment, as opposed to routine stent placement. The addition of intracranial stenting increases early stroke risk without providing superior stroke prevention over medical therapy, at least in the patient populations studied. In practical terms, this means that unless a patient is truly failing medical therapy (e.g., multiple recurrent strokes despite optimal management) and meets very strict criteria, endovascular stenting should be approached with great caution if at all.

### Limitations and Future Prospects

The overall quality of evidence in our review is moderate. All included studies were randomized controlled trials with outcome adjudication, which reduces bias. However, they were open-label; participants and treating physicians knew the assigned treatment, which could influence adjunctive care or reporting of minor events like TIA. We assume major endpoints like stroke and death were assessed objectively by blinded committees, as reported in the trials, mitigating detection bias. A limitation of the evidence is the trial heterogeneity noted above like differences in inclusion criteria (especially timing post-event), patient populations, and device types. We attempted to account for these differences with subgroup and meta-regression analyses; notably, no heterogeneity in the primary 1-year outcomes was detected statistically (I^2^ values were low). Still, with only four trials, the power to detect heterogeneity or subgroup effects is limited. Our meta-regression is constrained by the small number of studies (each covariate test had effectively 3 degrees of freedom), so while no significant modifiers emerged, one must be cautious in over-interpreting a null interaction since subtle differences could be missed. Patient-level meta-analysis would be ideal to explore subgroup effects with greater granularity (e.g., lesion location, degree of collaterals, etc.), but such data were not available for this review.

That said, our findings do not close the book on endovascular treatment for ICAS. Instead, they refine the focus: any future efforts should aim at improving safety and targeting those who stand to benefit most. The data from WEAVE and CASSISS show that with careful selection (delayed intervention, favorable lesion morphology, experienced operators), peri-procedural complication rates can be brought down substantially. The BASIS trial suggests that a strategy not involving permanent stent implantation might achieve a net benefit. Therefore, future research should explore these avenues. Ongoing and future trials might compare modern balloon angioplasty or newer intracranial stents (e.g., drug-eluting stents or flow-modulation devices) against best medical therapy, preferably in patients at particularly high risk of recurrence, such as those demonstrating hemodynamic compromise on imaging or evidence of cerebral hypoperfusion. Additionally, individual patient data meta-analyses could help pinpoint subgroups (perhaps defined by infarct pattern, collateral status, or genetic factors) that could benefit from revascularization. So far, no clear subgroup has emerged, but this question warrants further exploration with larger pooled datasets.

## 5. Conclusions

Our meta-analysis confirms that intracranial stenting does not offer a significant benefit over medical therapy in preventing strokes among patients with symptomatic ICAS, and it is associated with increased early procedural risks. This conclusion remains consistent across various randomized controlled trials, patient populations, and clinical contexts. The uniformity of findings across high-quality studies lends strong support for current clinical decision-making. Therefore, intensive medical management should remain the standard approach for most patients. Endovascular procedures should be limited to research settings or select rescue cases, unless future data demonstrates a more favorable risk–benefit profile.

## Figures and Tables

**Table 1:** Inclusion and exclusion criteria.

Inclusion Criteria	Exclusion Criteria
Randomized controlled trials (RCTs) Adult patients (≥18 years old) Diagnosed with symptomatic intracranial atherosclerotic stenosis (ICAS) with 50–99% stenosis severity Intervention: Endovascular treatment with stenting plus optimal medical therapy Comparator: Optimal medical therapy alone, including: - Dual antiplatelet therapy - Aggressive risk factor management Report at least one relevant clinical outcome: - Ischemic or hemorrhagic stroke - Transient ischemic attack (TIA) - Death - Restenosis - Adverse procedural events Minimum follow-up duration of one month Published in English	Non-randomized studies: - Observational studies - Case series - Case reports - Review articles Studies focusing exclusively on extracranial carotid stenosis Studies lacking sufficient outcome data for analysis Follow-up duration of less than one month

**Table 2:** Characteristics of the included studies and patients.

Authors	Country	Sample Size/Male/Female	Number of Patients (N)	Active Smokers (N)	Age (Mean ± SD, Years)	Stent Type in the Intervention Group (STN+MT)
			STN+MT/MT Alone	STN+MT/MT Alone	STN+MT/MT Alone	
Chimowitz et al., 2011 (SAMMPRIS) [13]	USA	451 (272/179)	224/227	54/69	61 ± 10.7/59.5 ± 11.8	Wingspan stent
Miao et al., 2012 [16]	China	70 (49/21)	31/39	21/19	53.42 ± 13.55/49.18 ± 9.29	Balloon Angioplasty: 4, Wingspan stent: 7, Coroflex blue: 17, PTA Firebird stent: 1
Zaidat et al., 2015 (VISSIT) [14]	International	111 (73/38)	58/53	11/12	61.8 ± 12.28/61.8 ± 12.28	Balloon-expanding stent
Gao et al., 2022 (CASSISS) [15]	China	358 (263/95)	176/182	41/50	56.7 ± 9.4/55.9 ± 9.8	Wingspan stent

STN: stenting; MT: medical therapy; SD: standard deviation.

**Table 3:** Baseline clinical and angiographic characteristics of participants.

Authors	Stroke as a Cause of the Intervention	TIA as a Cause of the Intervention	Arteries Affected		Time from Event to Randomization (Mean ± SD)	Degree of Arterial Stenosis (Mean ± SD)	Receiving Antithrombotic Before Event
	STN+MT/MT Alone	STN+MT/MT Alone	STN+MT	MT Alone	STN+MT/MT Alone	STN+MT/MT Alone	STN+MT/MT Alone
Chimowitz et al., 2011 (SAMMPRIS) [13]	142/152	82/75	ICA: 45 (20.1), MCA: 92 (41.1), Vertebral 38 (17), Basilar: 49 (21.9)	ICA: 49 (21.6), MCA: 105 (46.3), Vertebral: 22 (9.7), Basilar: 51 (22.5	9.0 ± 9.0/6.7 ± 3.7	80.0 ± 7.0/81.0 ± 7.0	145/141
Miao et al., 2012 [16]	7/8	29/26	Left MCA: 12, Right MCA: 24	Left MCA: 18, Right MCA: 16	NA	83.9 ± 8.1/85.0 ± 6.7	NA
Zaidat et al. 2015 (VISSIT) [14]	36/34	24/22	NA	NA	12.3 ± 9.6/15.2 ± 10.3	78.9 ± 7.3/80.4 ± 7.5	NA
Gao et al., 2022 (CASSISS) [15]	89/105	87/77	MCA (M1): 65 (36.9), Basilar artery: 50 (28.4), Intracranial vertebral artery: 46 (26.1), Intracranial internal carotid artery 15 (8.5)	MCA (M1): 79 (43.4), Basilar artery: 52 (28.6), Intracranial vertebral artery: 34 (18.7), Intracranial internal carotid artery 17 (9.3)	42.3 ± 28.8/44.0 ± 29.9	78.4 ± 6.4/76.9 ± 5.8	49/48

STN: stenting; MT: medical therapy; NA: records are non-available; SD: standard deviation.

**Table 4:** Results of the meta-regression analysis for selected outcomes.

Characteristic	Stroke at 30 Days			Stroke at 1 Year			Intracerebral Hemorrhage at 30 Days			Transient Ischemic Attack at 1 Year		
	Beta	95% CI	*p*-Value	Beta	95% CI	*p*-Value	Beta	95% CI	*p*-Value	Beta	95% CI	*p*-Value
Age	−0.02	−0.28, 0.23	0.852	0.17	−0.07, 0.40	0.161	0.05	−0.67, 0.77	0.887	−0.12	−0.33, 0.09	0.247
Year of publication	0.03	−0.12, 0.19	0.662	−0.08	−0.32, 0.15	0.499	−0.07	−0.43, 0.30	0.713	−0.01	−0.27, 0.24	0.917
Proportion of males	0.03	−0.09, 0.16	0.580	−0.06	−0.27, 0.14	0.531	−0.06	−0.37, 0.25	0.703	0.12	−0.22, 0.45	0.495
Smoking	0.00	−0.09, 0.09	0.982	−0.02	−0.11, 0.07	0.665	0.09	−0.51, 0.69	0.768	0.02	−0.03, 0.07	0.375
Stroke as a cause of intervention	−0.02	−0.08, 0.05	0.656	0.02	−0.06, 0.09	0.664	0.06	−0.29, 0.41	0.752	−0.02	−0.07, 0.02	0.345
TIA as a cause of intervention	0.02	−0.05, 0.09	0.575	−0.01	−0.09, 0.07	0.834	−0.08	−0.44, 0.29	0.673	0.02	−0.03, 0.07	0.379
Degree of arterial stenosis	−0.03	−0.49, 0.43	0.911	0.11	−0.40, 0.63	0.674	0.25	−1.14, 1.64	0.725	0.08	−0.22, 0.38	0.604

## Data Availability

The raw data supporting the conclusions of this article will be made available by the authors on request.

## Supplementary Materials

The following supporting information can be downloaded at: https://www.mdpi.com/article/10.3390/clinpract15060113/s1, File S1: Search Strategy.

## Abbreviations

The following abbreviations are used in this manuscript:

ICAS	Intracranial Atherosclerotic Stenosis
PTAS	Percutaneous Transluminal Angioplasty and Stenting
STN+MT	Stenting Plus Medical Therapy
MT	Medical Therapy
TIA	Transient Ischemic Attack
RCT	Randomized Controlled Trial
SAMMPRIS	Stenting and Aggressive Medical Management for Preventing Recurrent Stroke in Intracranial Stenosis
VISSIT	Vitesse Intracranial Stent Study for Ischemic Stroke Therapy
CASSISS	Chinese Angioplasty and Stenting for Symptomatic Intracranial Severe Stenosis
BASIS	Balloon Angioplasty vs. Best Medical Therapy in Symptomatic ICAS
WEAVE	Wingspan Stent System Post Market Surveillance Study
ICH	Intracerebral Hemorrhage
