# Supplementary material for: Comparing Stenting with Medical Therapy Versus Medical Therapy Alone in Patients with Intracranial Atherosclerotic Stenosis: A Current Systematic Review and Meta-Analysis
Source: Clin Pract. 2025 Jun 19;15(6):113. doi: 10.3390/clinpract15060113 (PMC12191942; doi:10.3390/clinpract15060113)
Supplement: Supplementary file 1 [file clinpract-15-00113-s001.zip › clinpract-3677697-supplementary.pdf]

clinics and practice

MDPI

File S1: Search Strategy.

Comparing Stenting with Medical Therapy versus Medical Therapy Alone in Patients with Intracranial Atherosclerotic Stenosis: A Current Systematic Review and Meta-Analysis

Khalid Bin Aziz [1,3], Hussam Alhathlol [1,3], Fahad Bin Aziz [1,3], Mohammed Alshammari [2,3], Mohammed Ali Alhefdhi [1,3], Abdulrahman M. Alrasheed [1,3], Nawwaf Alfayez [1,3] and Thamer S. Alhowaish [2,3]

1 College of Medicine, King Saud bin Abdulaziz University for Health Sciences, Riyadh, Saudi Arabia.
2 Department of Neurology, King Abdulaziz Medical City, Ministry of the National Guard Health Affairs (MNGHA), Riyadh, Saudi Arabia.
3 King Abdullah International Medical Research Center, Riyadh, Saudi Arabia.
* Correspondence: binazizkh@gmail.com

EBSCOhost Search Strategy via Saudi Digital Library (SDL):.

Step	Search String (EBSCO syntax)
#1	intracranial stenosis OR intracranial atherosclerosis OR cerebral artery stenosis
#2	intracranial stenting OR endovascular therapy OR angioplasty OR wingspan stent OR self-expanding stent
#3	medical therapy OR aggressive medical management OR dual anti platelet therapy OR aspirin and clopidogrel
#4	stroke OR ischemic stroke OR hemorrhagic stroke or Mortality OR restenosis
#5	randomized controlled trials OR RCT
#6	#1 AND #2 AND #3 AND #4 AND #5

Search was done on: 5 June 2025.

Scopus search strategy:

Step	Keywords / Search String (Scopus syntax)
#1	TITLE-ABS-KEY("intracranial stenosis" OR "intracranial atherosclerosis" OR "cerebral artery stenosis")
#2	TITLE-ABS-KEY("intracranial stenting" OR "endovascular therapy" OR "angioplasty" OR "wingspan stent" OR "balloon-expandable stent" OR "self-expanding stent")
#3	TITLE-ABS-KEY("medical therapy" OR "dual antiplatelet therapy" OR "aspirin and clopidogrel" OR "aggressive medical management")
#4	TITLE-ABS-KEY("stroke" OR "ischemic stroke" OR "hemorrhagic stroke" OR "mortality" OR "restenosis")
#5	TITLE-ABS-KEY("randomized controlled trial" OR "RCT")
#6	#1 AND #2 AND #3 AND #4 AND #5

Search was done on: 5 June 2025.

Web Of Science search strategy:.

Step	Search Terms (Web of Science Syntax: ALL=)
#1	ALL=(Intracranial stenosis OR Intracranial atherosclerosis OR Cerebral artery stenosis)
#2	ALL=(Intracranial stenting OR Endovascular therapy OR Angioplasty OR Wingspan stent OR Balloon-expandable stent OR Self-expanding stent)
#3	ALL=(Medical therapy OR Aggressive medical management OR Dual antiplatelet therapy OR Aspirin and Clopidogrel)
#4	ALL=(Stroke OR Ischemic stroke OR Hemorrhagic stroke OR Mortality OR Restenosis)
#5	ALL=(Randomized controlled trial OR RCT)
#6	#1 AND #2 AND #3 AND #4 AND #5

Search was done on: 3 March 2025.

Cochrane Library search strategy:.

Step	Search Terms
#1	Intracranial stenosis OR Intracranial atherosclerosis OR Cerebral artery stenosis
#2	Intracranial stenting OR Endovascular therapy OR Angioplasty OR Wingspan stent OR Balloon-expandable stent OR Self-expanding stent
#3	Medical therapy OR Aggressive medical management OR Dual antiplatelet therapy OR Aspirin and Clopidogrel
#4	Stroke OR Ischemic stroke OR Hemorrhagic stroke OR Mortality OR Restenosis
#5	Randomized controlled trial OR RCT
#6	#1 AND #2 AND #3 AND #4 AND #5

Search was done on: 3 March 2025.

PubMed search strategy:.

Step	Search Terms Used (PubMed Syntax)
#1	((("intracranial"[All Fields] OR "intracranially"[All Fields]) AND ("constriction, pathologic"[MeSH Terms] OR ("constriction"[All Fields] AND "pathologic"[All Fields]) OR "pathologic constriction"[All Fields] OR "stenosi"[All Fields] OR "stenosis"[All Fields])) OR ("intracranial arteriosclerosis"[MeSH Terms] OR ("intracranial"[All Fields] AND "arteriosclerosis"[All Fields]) OR "intracranial arteriosclerosis"[All Fields] OR ("intracranial"[All Fields] AND "atherosclerosis"[All Fields]) OR "intracranial atherosclerosis"[All Fields]) OR (("cerebral arteries"[MeSH Terms] OR ("cerebral"[All Fields] AND "arteries"[All Fields]) OR "cerebral arteries"[All Fields] OR ("cerebral"[All Fields] AND "artery"[All Fields]) OR "cerebral artery"[All Fields]) AND ("constriction, pathologic"[MeSH Terms] OR ("constriction"[All Fields] AND "pathologic"[All Fields]) OR "pathologic constriction"[All Fields] OR "stenosi"[All Fields] OR "stenosis"[All Fields]))) AND ((("intracranial"[All Fields] OR "intracranially"[All Fields])
#2	("stent s"[All Fields] OR "stentings"[All Fields] OR "stents"[MeSH Terms] OR "stents"[All Fields] OR "stent"[All Fields] OR "stented"[All Fields] OR "stenting"[All Fields])) OR ("Endovascular"[All Fields] AND ("therapeutics"[MeSH Terms] OR "therapeutics"[All Fields] OR "therapies"[All Fields] OR "therapy"[MeSH Subheading] OR "therapy"[All Fields] OR "therapy s"[All Fields] OR "therapys"[All Fields])) OR ("angioplastied"[All Fields] OR "angioplasty"[MeSH Terms] OR "angioplasty"[All Fields] OR "angioplasties"[All Fields]) OR (("wingspan"[All Fields] OR "wingspans"[All Fields]) AND ("stent s"[All Fields] OR "stentings"[All Fields] OR "stents"[MeSH Terms] OR "stents"[All Fields] OR "stent"[All Fields] OR "stented"[All Fields] OR "stenting"[All Fields])) OR ("Balloon-expandable"[All Fields] AND ("stent s"[All Fields] OR "stentings"[All Fields] OR "stents"[MeSH Terms] OR "stents"[All Fields] OR "stent"[All Fields] OR "stented"[All Fields] OR "stenting"[All Fields])) OR ("Self-expanding"[All Fields] AND ("stent s"[All Fields] OR "stentings"[All Fields] OR "stents"[MeSH Terms] OR "stents"[All Fields] OR "stent"[All Fields] OR "stented"[All Fields] OR "stenting"[All Fields])))
#3	((("medic"[All Fields] OR "medical"[All Fields] OR "medicalization"[MeSH Terms] OR "medicalization"[All Fields] OR "medicalizations"[All Fields] OR "medicalize"[All Fields] OR "medicalized"[All Fields] OR "medicalizes"[All Fields] OR "medicalizing"[All Fields] OR "medically"[All Fields] OR "medicals"[All Fields] OR "medicated"[All Fields] OR "medication s"[All Fields] OR "medics"[All Fields] OR "pharmaceutical preparations"[Supplementary Concept] OR "pharmaceutical preparations"[All Fields] OR "medication"[All Fields] OR "pharmaceutical preparations"[MeSH Terms] OR ("pharmaceutical"[All Fields] AND "preparations"[All Fields]) OR "medications"[All Fields]) AND ("therapeutics"[MeSH Terms] OR "therapeutics"[All Fields] OR "therapies"[All Fields] OR "therapy"[MeSH Subheading] OR "therapy"[All Fields] OR "therapy s"[All Fields] OR "therapys"[All Fields])) OR (("aggress"[All Fields] OR "aggressed"[All Fields] OR "aggressing"[All Fields] OR "aggression"[MeSH Terms] OR "aggression"[All Fields] OR "aggressions"[All Fields] OR "aggressive"[All Fields] OR "aggressiveness"[All Fields] OR "aggressively"[All Fields] OR "aggressives"[All Fields] OR "aggressivity"[All Fields]) AND ("medic"[All Fields] OR "medical"[All Fields] OR "medicalization"[MeSH Terms] OR "medicalization"[All Fields] OR "medicalizations"[All Fields] OR "medicalize"[All Fields] OR "medicalized"[All Fields] OR "medicalizes"[All Fields] OR "medicalizing"[All Fields] OR "medically"[All Fields] OR "medicals"[All Fields] OR "medicated"[All Fields] OR "medication s"[All Fields] OR "medics"[All Fields] OR "pharmaceutical

	preparations"[Supplementary Concept] OR "pharmaceutical preparations"[All Fields] OR "medication"[All Fields] OR "pharmaceutical preparations"[MeSH Terms] OR ("pharmaceutical"[All Fields] AND "preparations"[All Fields]) OR "medications"[All Fields]) AND ("manage"[All Fields] OR "managed"[All Fields] OR "management s"[All Fields] OR "managements"[All Fields] OR "manager"[All Fields] OR "manager s"[All Fields] OR "managers"[All Fields] OR "manages"[All Fields] OR "managing"[All Fields] OR "managment"[All Fields] OR "organization and administration"[MeSH Terms] OR ("organization"[All Fields] AND "administration"[All Fields]) OR "organization and administration"[All Fields] OR "management"[All Fields] OR "disease management"[MeSH Terms] OR ("disease"[All Fields] AND "management"[All Fields]) OR "disease management"[All Fields])) OR ("Dual"[All Fields] AND ("antiplatelet"[All Fields] OR "antiplatelets"[All Fields]) AND ("therapeutics"[MeSH Terms] OR "therapeutics"[All Fields] OR "therapies"[All Fields] OR "therapy"[MeSH Subheading] OR "therapy"[All Fields] OR "therapy s"[All Fields] OR "therapys"[All Fields])) OR (("aspirin"[Supplementary Concept] OR "aspirin"[All Fields] OR "aspirin"[MeSH Terms] OR "aspirins"[All Fields] OR "aspirin s"[All Fields] OR "aspirine"[All Fields]) AND ("clopidogrel"[Supplementary Concept] OR "clopidogrel"[All Fields] OR "clopidogrel"[MeSH Terms] OR "clopidogrel s"[All Fields])))
**#4**	("stroke"[MeSH Terms] OR "stroke"[All Fields] OR "strokes"[All Fields] OR "stroke s"[All Fields] OR ("ischemic stroke"[MeSH Terms] OR ("ischemic"[All Fields] AND "stroke"[All Fields]) OR "ischemic stroke"[All Fields]) OR ("hemorrhagic stroke"[MeSH Terms] OR ("hemorrhagic"[All Fields] AND "stroke"[All Fields]) OR "hemorrhagic stroke"[All Fields]) OR ("mortality"[MeSH Terms] OR "mortality"[All Fields] OR "mortalities"[All Fields] OR "mortality"[MeSH Subheading]) OR "Restenosis"[All Fields])
**#5**	("randomized controlled trial"[Publication Type] OR "randomized controlled trials as topic"[MeSH Terms] OR "randomized controlled trial"[All Fields] OR "randomised controlled trial"[All Fields] OR "RCT"[All Fields])
**#6**	#1 AND #2 AND #3 AND #4 AND #5

Search was done on: 3 March 2025.

Embase search strategy:.

Step	Keywords / EMTREE Terms Used
**#1**	'intracranial stenosis'/exp OR 'intracranial stenosis' OR 'intracranial atherosclerosis'/exp OR 'intracranial atherosclerosis' OR 'cerebral artery stenosis'/exp OR 'cerebral artery stenosis'
**#2**	'intracranial stenting'/exp OR 'intracranial stenting' OR 'endovascular therapy'/exp OR 'endovascular therapy' OR 'angioplasty'/exp OR 'angioplasty' OR 'wingspan stent'/exp OR 'wingspan stent' OR 'balloon-expandable stent' OR 'self-expanding stent'/exp OR 'self-expanding stent'
**#3**	'medical therapy'/exp OR 'medical therapy' OR 'dual antiplatelet therapy'/exp OR 'dual antiplatelet therapy' OR 'aspirin and clopidogrel' OR 'aggressive medical management'
**#4**	'stroke'/exp OR 'stroke' OR 'ischemic stroke'/exp OR 'ischemic stroke' OR 'hemorrhagic stroke'/exp OR 'hemorrhagic stroke' OR 'mortality'/exp OR 'mortality' OR 'restenosis'/exp OR 'restenosis'
**#5**	'randomized controlled trial'/exp OR 'randomized controlled trial' OR 'rct'
**#6**	#1 AND #2 AND #3 AND #4 AND #5
**#7**	#6 AND **'randomized controlled trial'**/de

Search was done on: 5 June 2025.
